# Changes in the Rate of Emergency Presentation in Patients with Functional Neurological Disorder Attending a Long-term Community Care Program for FND

**DOI:** 10.1192/j.eurpsy.2023.1152

**Published:** 2023-07-19

**Authors:** M. Gheis, G. Sekhon

**Affiliations:** 1 Psychiatry, University of British Columbia, Victoria, Canada; 2 Psychiatry, Nassau University Medical Center, New York, United States

## Abstract

**Introduction:**

Patients with Functional Neurological Disorder have a high return rate to Emergency Rooms.

**Objectives:**

To assess possible changes in Emergency Room presentation rates in patients with Functional Neurological Disorder following their attendance of specialized long-term multidisciplinary treatment and rehabilitation program.

**Methods:**

Seventy-two adult patients with Functional Neurological Disorder were included. These patients were consecutive referrals accepted for ongoing specialist FND treatment.

The total number of Emergency Room presentations in the year prior to program admission was obtained from central health records. Patients were provided ongoing treatment for one year, during which the number of ER presentations was monitored. Patients received one or more of the following treatment modalities: psychoeducation, psychological therapy, psychologically informed physical and occupational rehabilitation and psychopharmacological treatments.

We subsequently compared high and low emergency service users. Low ER users are those with pre-treatment Emergency Room presentations of less than 3 per year. High emergency service users are those who presented to the emergency room 3 or more times per year before the start of their treatment.

**Results:**

The mean emergency room presentation per year in the year leading to patients referral was 2.6 per patient, SD 9.4; dropped to 1.2 emergency room presentations per year, with a standard deviation of 4.4 in the year following the start of treatment. The difference was statistically significant (p= 0.02).

There was a strong positive correlation between the pre and post-treatment number of presentations with a Pearson Correlation Coefficient of 0.976 (95% Confidence Interval 0.962 to 0.985).

The reduction in emergency room presentations in both high and low-emergency service user groups was significant, with a mean difference of 12 ER visits a year in high-frequency emergency service users (p= 0.04) and a mean difference of 0.5 visits a year in low-frequency emergency service users (p < 0.001).

**Conclusions:**

Ongoing specialist treatment and rehabilitation of patients with Functional Neurological Disorder significantly reduce their need for emergency room presentation, regardless of the treatment modality.

**Disclosure of Interest:**

None Declared

